# The Long Life Family Study: Sequencing Exceptional Pedigrees for Rare Protective Variants

**DOI:** 10.1093/geroni/igaa057.3127

**Published:** 2020-12-16

**Authors:** Michael Province, Kaare Christensen, Stephanie Consentino, Joseph Lee, Anne Newman, Thomas Perls, Bharat Thyagarajan, Joseph Zmuda

**Affiliations:** 1 Washington University School of Medicine, SAINT LOUIS, Missouri, United States; 2 University of Southern Denmark, Odense C, Syddanmark, Denmark; 3 Columbia University, New York, New York, United States; 4 University of Pittsburgh, Pittsburgh, Pennsylvania, United States; 5 Boston University School of Medicine, Boston, Massachusetts, United States; 6 University of Minnesota, Minneapolis, Minnesota, United States

## Abstract

The Long Life Family Study (LLFS) has longitudinally measured key aging phenotypes on 4,953 participants (539 pedigrees) in the USA and Denmark selected for exceptional familial longevity. On average, both generations of the LLFS sample are healthier than average for their age/sex, for many phenotypes. However, the pedigrees are heterogeneous, with different families showing familial clustering of protection for different phenotypes. Linkage analyses identified extremely strong genetic linkage peaks for many cross-sectional as well as longitudinal trajectory rates of change phenotypes. These peaks are NOT explained by GWAS SNPs (either measured or imputed). Pedigree specific HLODs and preliminary deep sequencing suggests that these peaks are driven by rare, protective variants running in selected pedigrees. Whole Genome Sequencing, a third longitudinal visit, and extensive OMICs (transcriptomics, epigenomics, metabolomics and proteomics) will help us resolve the mechanisms behind these protective genetically linked variants, and could illuminate new biology and enable new therapeutics.

